# Switchable Control of Scaffold Protein Activity via Engineered Phosphoregulated Autoinhibition

**DOI:** 10.1021/acssynbio.2c00122

**Published:** 2022-06-29

**Authors:** Arjan Hazegh Nikroo, Lenne J. M. Lemmens, Tim Wezeman, Christian Ottmann, Maarten Merkx, Luc Brunsveld

**Affiliations:** Laboratory of Chemical Biology, Department of Biomedical Engineering and Institute for Complex Molecular Systems, Technische Universiteit Eindhoven, Den Dolech 2, Eindhoven, 5612AZ Arizona, The Netherlands

**Keywords:** scaffold proteins, protein engineering, auto-regulation, 14-3-3, synthetic signaling

## Abstract

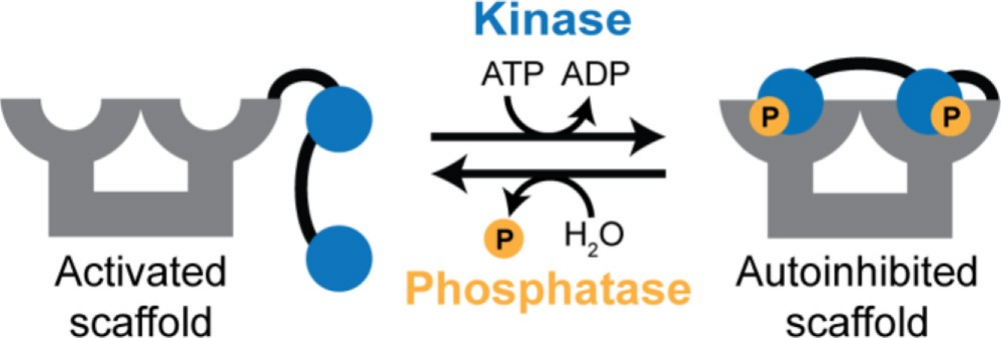

Scaffold proteins operate as organizing hubs to enable high-fidelity signaling, fulfilling crucial roles in the regulation of cellular processes. Bottom-up construction of controllable scaffolding platforms is attractive for the implementation of regulatory processes in synthetic biology. Here, we present a modular and switchable synthetic scaffolding system, integrating scaffold-mediated signaling with switchable kinase/phosphatase input control. Phosphorylation-responsive inhibitory peptide motifs were fused to 14-3-3 proteins to generate dimeric protein scaffolds with appended regulatory peptide motifs. The availability of the scaffold for intermolecular partner protein binding could be lowered up to 35-fold upon phosphorylation of the autoinhibition motifs, as demonstrated using three different kinases. In addition, a hetero-bivalent autoinhibitory platform design allowed for dual-kinase input regulation of scaffold activity. Reversibility of the regulatory platform was illustrated through phosphatase-controlled abrogation of autoinhibition, resulting in full recovery of 14-3-3 scaffold activity.

## Introduction

Signaling pathways are essential biological components that elegantly regulate cellular processes with high spatiotemporal control. A key aspect of synthetic biology is the bottom-up construction of such signaling pathways with built-in features that allow monitoring and modulation of the underlying processes to elucidate the working principles of cell signaling.^1−3^ Rewiring pathways or even constructing fully artificial signaling systems offers compelling opportunities with therapeutic, diagnostic, and industrial applications.^4^ Natural signaling pathways frequently rely on enzyme-controlled covalent modifications and the recruitment and activation of proteins on scaffold proteins.^5−7^ However, to date, only a few bottom-up synthetic protein-based signaling networks that incorporate a combination of such features have been reported, mainly because of a lack of robust scaffold protein platforms. Scaffold proteins are particularly of interest as these serve as organizing elements that assemble two or more signaling proteins without requiring additional cellular compartmentalization and with excellent spatiotemporal control.^8−11^ Within the context of synthetic biology, engineered scaffolds have provided substantial insight into the plasticity and molecular mechanisms of cell signaling. For instance, synthetic variants of well-characterized scaffold Ste5 have highlighted scaffold modularity via domain recombination, enabling pathways to respond to non-native inputs,^12^ the redirection of input–output responses between pathways,^13^ and the reshaping of complex response behaviors.^14^ In addition, synthetic scaffolds can be applied to optimize pathway function, as exemplified by engineered protein scaffolds that regulated enzyme stoichiometry to enhance the production rate of biomolecules relative to the native pathway.^15−18^

A major class of eukaryotic scaffold proteins is the 14-3-3 proteins. Members of this well-conserved protein family typically exist as constitutive homo- or heterodimers composed of different isoforms,^19−21^ with each monomer featuring an amphipathic binding groove that enables specific binding to phosphorylated serine or threonine binding motifs in target proteins. 14-3-3 proteins play crucial roles in facilitating proximity-driven protein–protein interactions (PPIs) and serve as regulatory tools in a wide variety of cellular processes.^19,21−24^ Recently, we reported the development of versatile molecular hubs by coupling phosphorylation-independent inhibitory peptide motifs to 14-3-3 scaffold proteins via protease-cleavable peptide linkers.^25^ While such protease-activatable scaffolds represent modular platforms that can be tuned to fit various applications, protease action is typically irreversible. Previous studies have also shown the flexibility of 14-3-3 toward the creation of protein assembly platforms that rely on the binding of specific phosphorylated peptide motifs, which, for instance, enabled the development of modular 14-3-3-based kinase sensors.^26−28^ In this work, we present a strategy to regulate the 14-3-3 scaffolding activity using reversible enzyme-controlled phosphorylation and dephosphorylation of covalently fused regulatory peptide modules. By integrating two key signaling mechanisms, scaffold-mediated signaling and phosphorylation, this approach allows for the bidirectional regulation of 14-3-3 scaffolding activity.

## Scaffold Design and Synthesis

**Figure 1:**
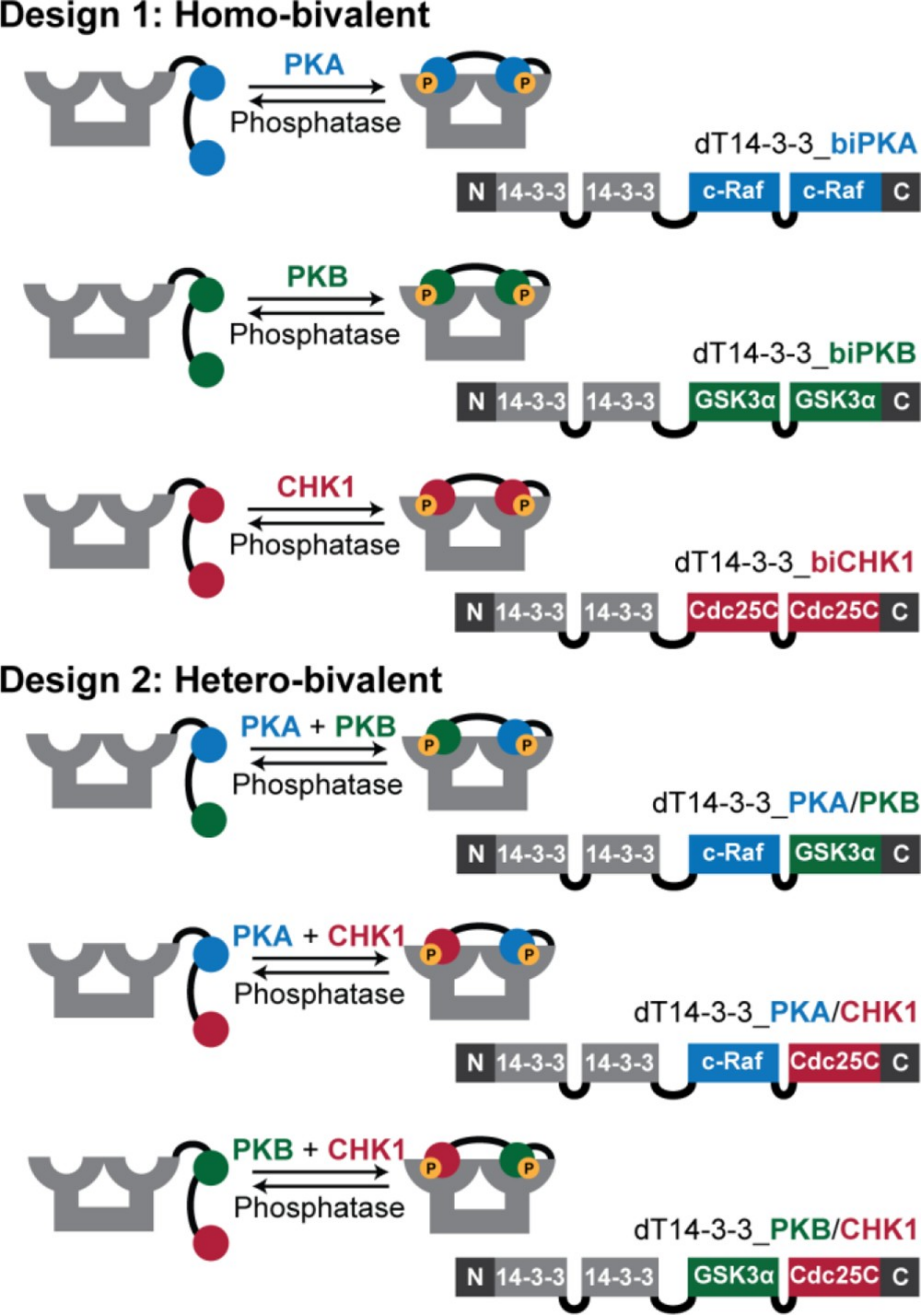
Schematic representation of phosphoregulated 14-3-3 scaffold designs and domain composition. Homo-bivalent design features two recognition motifs for the same kinase, separated by a peptide linker. Hetero-bivalent design features two orthogonal kinase recognition motifs to enable dual-input regulation using two distinct kinases.

The 14-3-3 scaffold platforms were designed via a modular approach, building on the protein architecture of previously developed protease-activatable 14-3-3 scaffolds.^25^ As scaffold chassis, two Tobacco 14-3-3 monomers were covalently linked via a (Gly-Gly-Ser)_10_ linker (dimeric Tobacco 14-3-3 scaffold, abbreviated as dT14-3-3) to prevent the formation of a mixture of different dimers.^22,27^ The inhibitory peptides were covalently fused to the C-terminal domain using a flexible peptide linker containing a TEV protease cleavage site.^25^ As such, two inherently asymmetric designs were investigated to explore the phosphoregulated autoinhibition of the dT14-3-3 scaffold. The homo-bivalent design (Figure 1) features two of the same inhibitory peptide motifs, enabling reversible blocking of 14-3-3’s binding grooves via one kinase signal. Conversely, the hetero-bivalent design (Figure 1) takes advantage of 14-3-3’s ability to bind motifs phosphorylated by different kinases by featuring two distinct kinase-responsive inhibitory motifs. Besides enabling reversible blocking, this design opens up opportunities for dual-input control and logic operations such as AND-gating to tune regulation specificity.

The inhibitory peptide motifs were derived from three 14-3-3 binding partners known to be phosphorylated by protein kinase A (PKA), protein kinase B (PKB), or checkpoint kinase 1 (CHK1).^26^ PKA phosphorylation of Raf proto-oncogene serine/threonine kinase (c-Raf)^29^ at residues serine-233 and serine-259 centers on the well-defined PKA recognition motif (R-R-X-S/T-Y),^30^ known to promote 14-3-3 binding.^31^ This bivalent peptide c-Raf sequence has indeed been shown to bind two 14-3-3 binding grooves simultaneously (Figure S2).^32,33^ The peptide sequence responsive to PKB was derived from glycogen synthase kinase-3α (GSK3α),^34^ featuring its PKB phosphorylation site serine-21. This peptide includes the PKB recognition motif (R-X-R-X-X-S),^35^ with 14-3-3 binding supported by binding prediction software.^24^ The peptide motif for CHK1 was derived from phosphatase cell-division-cycle 25C (Cdc25C), known to bind to 14-3-3 upon the phosphorylation of its serine-216 residue by CHK1.^36−38^ This peptide includes the CHK1 recognition motif (R-X-X-S/T)^39−42^ and adjacent residues hypothesized to be important for binding based on the crystal structure of the Cdc25C/14-3-3 interaction (Figure S3).^43^ Bivalent peptides for CHK1 and PKB were designed by linking two peptide motifs using a flexible 11 residue Gly-Gly-Ser linker, devised to span the distance between both binding grooves.^25^ Analogously, three hetero-bivalent peptides (PKA/CHK1, PKA/PKB, and PKB/CHK1) were designed by combining the singular recognition motifs using the same flexible linker (see Table S1 for details).

To evaluate the suitability of the inhibitory motifs, FITC-labeled peptides, representing the individual peptide motifs, were synthesized (See Figure S4, Tables S1 and S2 for details) and fluorescence polarization (FP) titration experiments were performed to assess their *inter*molecular binding to dT14-3-3. Scaffold titrations to these phosphorylated monovalent peptides revealed PKA259 and PKA233 to be the strongest binders (*K*_d_ values of 2.7 ± 0.1 μM and 8.0 ± 0.4 μM, respectively), whereas the phosphorylated CHK1 and PKB peptides displayed 100- and 1000-fold weaker binding than PKA259, respectively (Figure S5).

The potential for *intra*molecular binding of the peptides sequences attached to the 14-3-3 scaffold was inferred from the linker architecture and the effective concentration (*C*_eff_) it enforces. Because GGS-linker behavior can be described by the wormlike chain model,^44,45^*C*_eff_ could be derived from its predicted linker-dependent distance distribution and the distance that has to be spanned to reach the binding groove. The crystal structure of T14-3-3c (Figure S6) revealed the distance between residue R136 (a key coordinator of phosphorylated moieties)^28^ and the C-terminus of T14-3-3, to be ∼20 Å. Combined, the 71 residue linker between the C-terminus of T14-3-3 and the phosphoregulatory motif was calculated to yield a *C*_eff_ of ∼1 mM.

Plasmids were generated encoding for the dimeric 14-3-3 scaffolds fused to their inhibitory peptide motifs. For clarity and consistency, each protein construct is referred to by its corresponding kinases (e.g., the dT14-3-3 construct featuring a regulatory peptide with two sites responsive for PKA is abbreviated as dT14-3-3_biPKA). The engineered 14-3-3 scaffold constructs were expressed in *E. coli* BL21 (DE3) cells and purified by Ni^2+^-affinity chromatography using their *N*-terminal His_6_-tags. The purity of the recombinant proteins was assessed using sodium dodecyl sulfate-polyacrylamide gel electrophoresis (SDS-PAGE) (Figure S7) and quadrupole time-of-flight liquid chromatography–mass spectroscopy (Q-ToF LC–MS) (Figures S8 and S9). All proteins were obtained with excellent yields of ≥30 mg/L culture medium. While the acquisition of reliable MS data proved challenging due to broad chromatogram peaks and poorly distinguishable ion peak distributions, the anticipated molecular weights could be identified successfully after TEV protease-mediated cleavage of the linker that connects dT14-3-3 to the inhibitory peptide motifs (Figures S10–S12). Binding affinity studies using FP titration experiments with phosphorylation-independent 14-3-3 binding partner mExoS (derived from the C-terminal region of exoenzyme S ^420^QGLLDALDLAS^430^)^46^ and its high affinity bivalent (biExoS) variant^20,25^ (see Table S3 for details) showed that the covalently linked peptide motifs in their unphosphorylated state do not impede normal dT14-3-3 binding (Figure S13).

## Phosphorylation Regulates the Scaffolding Function of 14-3-3

**Figure 2:**
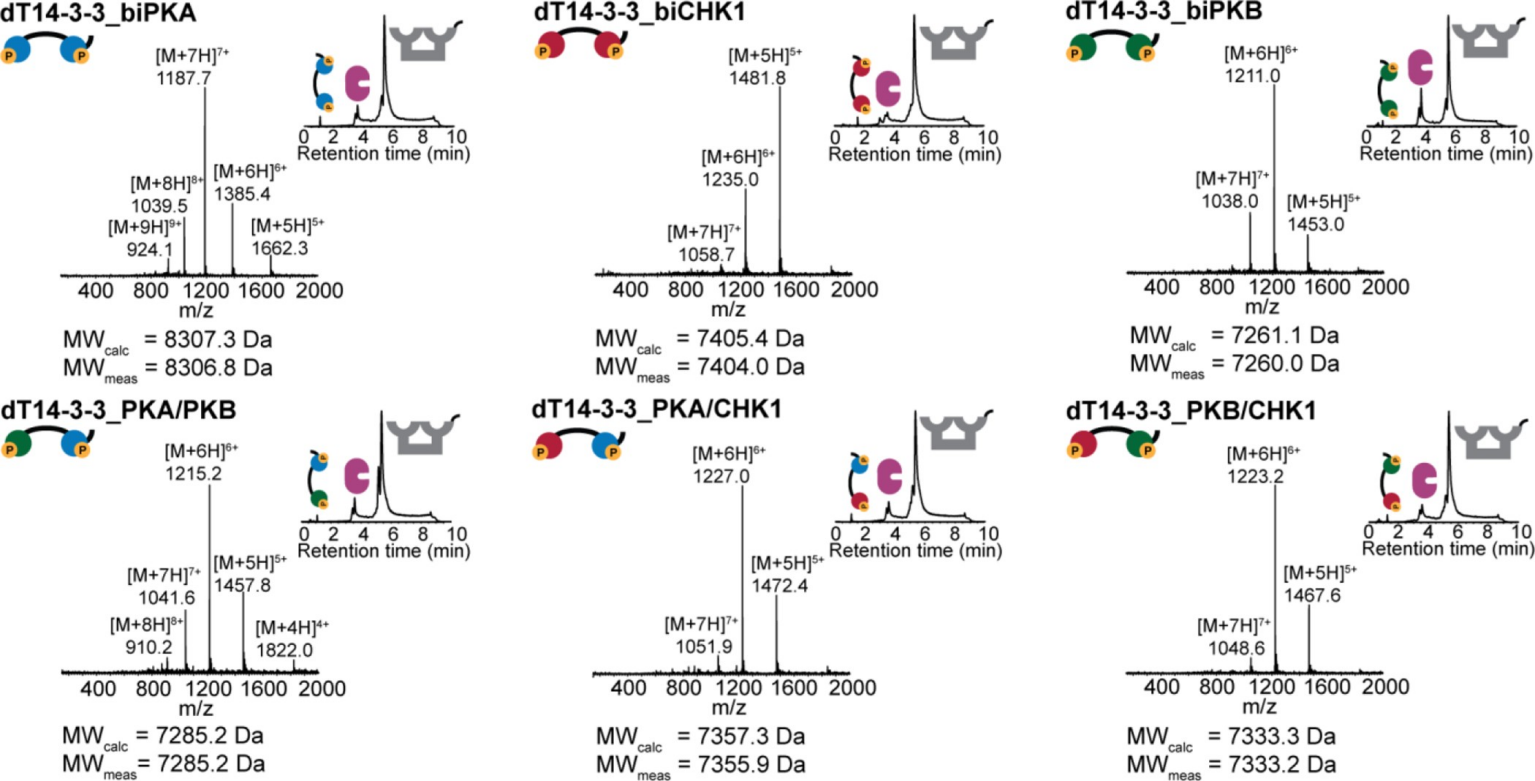
Q-Tof LC–MS analysis of dT14-3-3 constructs after phosphorylation. *m*/*z* spectra of the doubly phosphorylated peptide motifs are shown with the total ion count chromatograms (inlay) and corresponding calculated and measured molecular weights. Samples were treated with TEV protease (schematically depicted in pink) to enable a clear distinction of the phosphorylated peptide sequence.

Incubation of the dT14-3-3 scaffolds with their corresponding kinases and ATP led to double phosphorylation of all regulatory peptide motifs. This was confirmed via Q-ToF LC–MS analysis, which showed ion peak distributions corresponding to double phosphorylation (mass increase of +160 Da) (Figures 2 and S14). To show that the double phosphorylation indeed occurred on the peptide motifs and not on the scaffold backbone, TEV protease was used to sever the connection between the two constructs before LC–MS analysis.

**Figure 3:**
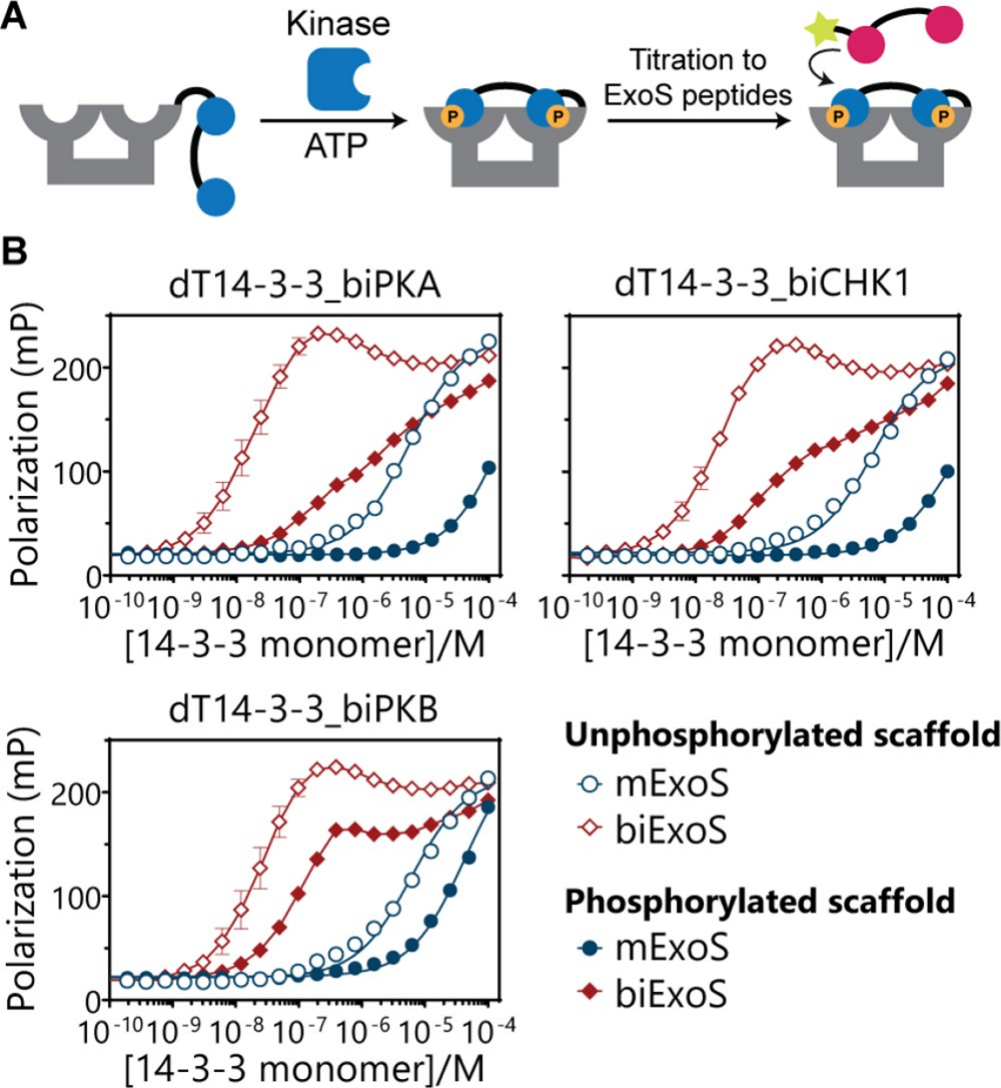
Fluorescence polarization competition assays of homo-bivalent dT14-3-3 constructs in unphosphorylated and phosphorylated states with mExoS and biExoS. (A) Schematic overview of phosphorylation-induced autoinhibition of the dT14-3-3 scaffold. (B) Titration of phosphorylated and unphosphorylated dT14-3-3_biPKA, dT14-3-3_biCHK1, and dT14-3-3_biPKB (95 pM–100 μM 14-3-3 monomer) to FITC-labeled ExoS peptides (15 nM). Datapoints for mExoS are fitted using eq 1. Datapoints for biExoS are connected by a line to guide the eye. Error bars represent SD (*n* = 3).

The level of autoinhibition imposed upon phosphorylation of the covalently attached inhibitory peptide motifs was assessed using FP binding assays (Figure 3, blue curves). It was anticipated that phosphorylation of the scaffold would induce *intra*molecular binding of the inhibitory peptide sequences to dT14-3-3, thus impeding the *inter*molecular binding of other 14-3-3 binding partners. Two ExoS peptides, as described above, were used as molecular probes to report on the effectiveness of the scaffold regulation process, while the homo-bivalent scaffold constructs (dT14-3-3_biPKA, dT14-3-3_biCHK1, and dT14-3-3_biPKB) were selected for detailed evaluation. Following phosphorylation of the different scaffold proteins, the low-affinity mExoS peptide showed up to 35-fold decrease in binding affinity relative to the unphosphorylated state of the dT14-3-3 scaffolds. A clear trend in the autoinhibition level was observed for the three scaffolds (from 35-fold decrease for dT14-3-3_biPKA to 10-fold for dT14-3-3_biPKB), which can be attributed to the differences in the binding affinity of the attached peptide motifs.^33,43^

Binding studies with the high-affinity biExoS peptide provide an additional stringent test, given its binding affinity in the low nanomolar range. Also for this peptide, the binding curves shifted to higher concentration upon phosphorylation of the 14-3-3 scaffolds (Figure 3, red curves). The biExoS titration curves for the phosphorylated scaffolds exhibited an unanticipated gradual increase in polarization with no upper plateau, rather than typical S-curves. Although these curve shapes prevent the determination of exact *K*_d_ values, a clear correlation between the binding affinity of the inhibitory peptide sequence and the extent of intramolecular blocking was observed. It should be noted that in this assay format, a small fraction of nonphosphorylated dT14-3-3 protein will still lead to a respectable remaining binding affinity. The presence of such minor quantities is likely and thus only further testifies to the effective inhibition by the phosphorylation event.

## Switching of 14-3-3 Scaffold Activity

**Figure 4:**
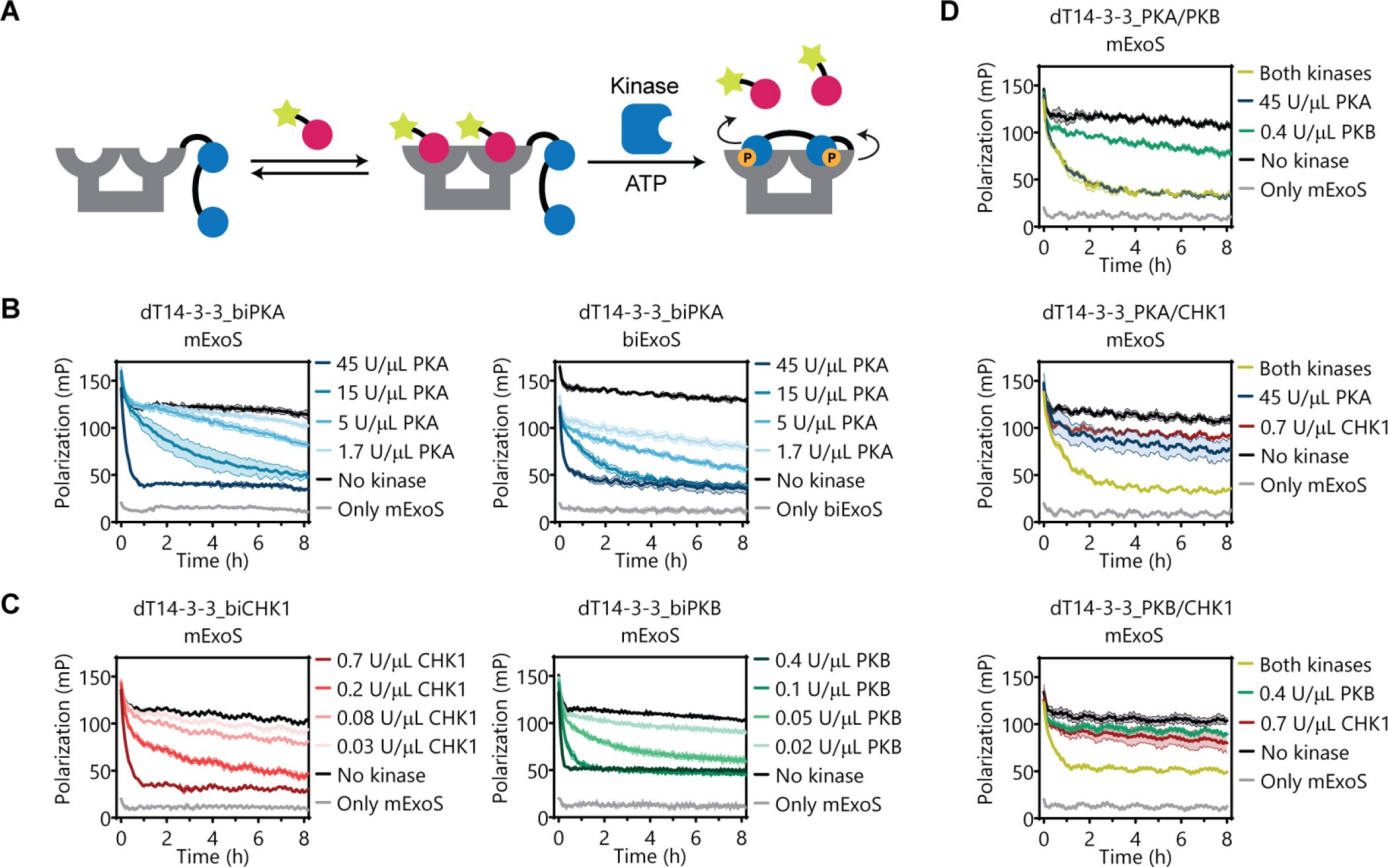
Kinetic fluorescence polarization assays of dT14-3-3 scaffold constructs in the presence of kinases. (A) Schematic representation of the kinetic assay format. Following the incubation of the 14-3-3 scaffold with the labeled ExoS peptide, kinase is added to phosphorylate the inhibitory peptide motif, inducing the displacement of the ExoS peptide. (B–D) Kinetic FP assays were performed by incubating the dT14-3-3_inhibitory peptide scaffolds (10 μM 14-3-3 monomer for mExoS, 40 nM 14-3-3 monomer for biExoS), 500 μM ATP, and 15 nM FITC-labeled ExoS peptide with corresponding kinases PKA (0–45 U/μL), CHK1 (0–0.7 U/μL), or PKB (0–0.4 U/μL). The hetero-bivalent scaffolds were incubated with the highest concentrations of the corresponding kinases separately, as a well as with both kinases simultaneously. Control (gray) shows ExoS peptide only. Error clouds represent SD (*n* = 3). The initial decrease in polarization observed for all measurements results from temperature equilibration at 30 °C.

A kinetic FP assay was performed to demonstrate that the phosphorylation status can be employed to regulate 14-3-3 scaffold availability via kinase-dependent autoinhibition (Figure 4A). Here, the unphosphorylated dT14-3-3_biPKA scaffold (selected for its clear difference between the unphosphorylated and phosphorated state) and the two FITC-labeled ExoS peptides were incubated with varying kinase concentrations at 30 °C, while monitoring the degree of autoinhibition over time (Figure 4B). The concentration of dT14-3-3_biPKA was chosen such that the largest fold-decrease in the FP signal between the nonphosphorylated and phosphorylated states could be observed, while only permitting minimal binding of the ExoS peptide in the phosphorylated state of the scaffold (for mExoS [dT14-3-3_biPKA] = 10 μM, for biExoS [dT14-3-3_biPKA] = 40 nM). The concentrations of the kinases were chosen such as to allow for rather slow modification rates to enable easy comparative detection of phosphorylation and effects on the resulting 14-3-3 scaffolding properties. The kinetic measurements revealed unambiguous decreases in the FP signal over time for both ExoS peptides upon the addition of kinase, supporting the hypothesis that phosphorylation of the covalently attached inhibitory peptide by PKA induces a gradual decrease in scaffold availability. The incubation of scaffold in the presence of high PKA concentrations (45 U/μL) yielded rapid decreases in the FP signal, indicative of rapid phosphorylation. As expected, significantly longer incubation times were required to reach full phosphorylation at low kinase concentrations, demonstrating that the amount and rate of autoinhibition can be tuned by the kinase activity. Analogous evaluation of the dT14-3-3_biCHK1 and dT14-3-3_biPKB scaffolds revealed similar tunable autoinhibitory behavior, logically at different kinase concentration (Figure 4C). The slightly weaker extent of functional autoinhibition enforced by the phosphorylated PKB motif, relative to the PKA and CHK1 motifs, corresponds with its lower 14-3-3 binding affinity. Collectively, these results illustrate that the scaffold activity can be switched by phosphorylation, that this switching is independent of prior binding of interaction partners, and that this phosphorylation-induced autoinhibition can be imposed at distinct concentration regimes.

Inspired by the results acquired for single kinase input regulation, we next explored the potential for dual-input regulation by evaluating the hetero-bivalent scaffolds with a similar time-resolved assay format. The incubation of dT14-3-3_PKA/PKB with PKA and PKB induced a comparable level of autoinhibition as for the homo-bivalent scaffolds (Figure 4D). The addition of solely PKB to dT14-3-3_PKA/PKB induced only a minor autoinhibitory effect, testifying to the need of double phosphorylation for full scaffold inhibition. Incubation with PKA alone yielded a full autoinhibitory response. This can most probably be attributed to the cross-reactivity of PKA toward PKB recognition motifs.^47^ The dT14-3-3_PKA/CHK1 and dT14-3-3_PKB/CHK1 scaffolds, in contrast, showed a clear dual input requirement for both kinases. The absence of kinase cross-reactivity for these hetero-bivalent motifs makes them most suited as dual input autoinhibition platforms. The addition of either kinase alone resulted in only a partial blockage of dT14-3-3, with slight differences in autoinhibitory effect in line with the trend in 14-3-3 binding affinities observed for the phosphorylated peptide motifs.

**Figure 5:**
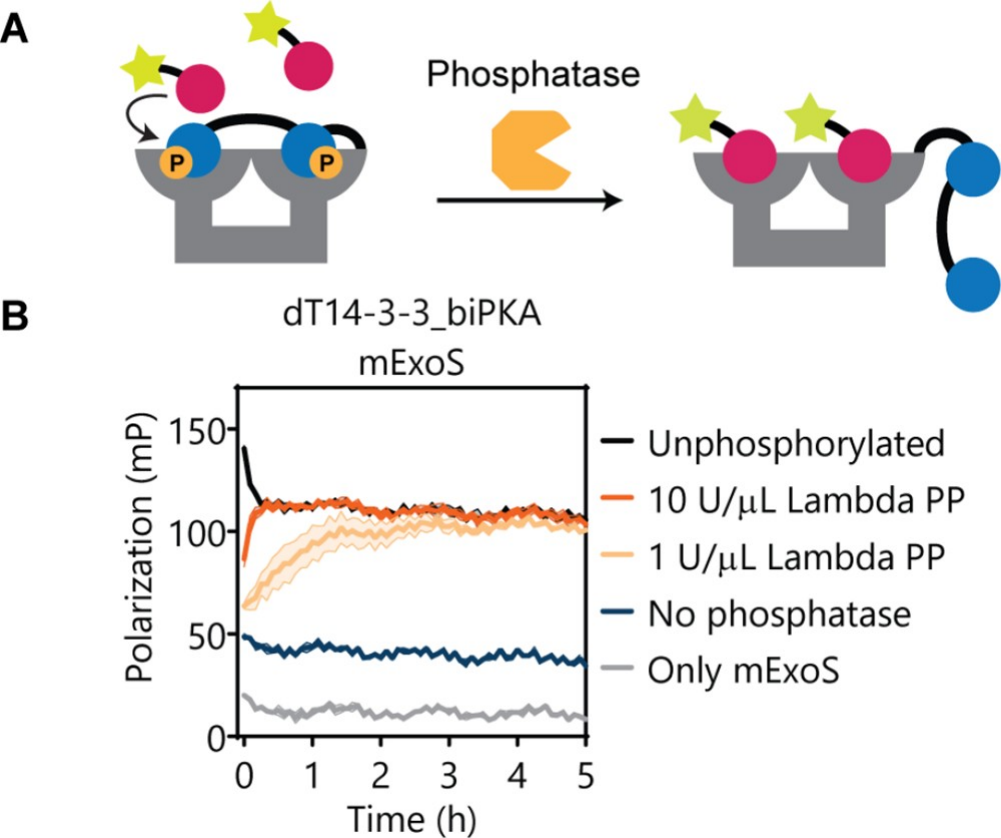
Recovery of 14-3-3 scaffold activity by dephosphorylation. (A) Schematic representation of the dephosphorylation assay. (B) Phosphorylated dT14-3-3_biPKA (10 μM 14-3-3 monomer) and 15 nM FITC-labeled mExoS were incubated in the presence and absence of varying concentrations of Lambda PP (0–10 U/μL). In the control (gray), only mExoS peptide is incubated. Error clouds represent SD (*n* = 3).

Reversible regulation of scaffold activity was generated by the addition of a phosphatase. It was hypothesized that dephosphorylation of the inhibitory peptide motifs would liberate the 14-3-3 binding groove, making it again available for intermolecular binding partners. As such, first, the dT14-3-3_biPKA scaffold was fully phosphorylated using PKA, enforcing maximum autoinhibition of the 14-3-3 scaffold. Subsequently, PKA was blocked by the addition of the inhibitor H89, and Mn^2+^-dependent phosphatase Lambda PP and FITC-labeled mExoS were added to monitor the dephosphorylation of the inhibitory peptide motifs over time using FP (Figure 5). We selected for a broadly active phosphatase to represent the, typically more, nonspecific dephosphorylation mechanisms as compared to the more selective phosphorylation by kinases. The addition of 10 U/μL Lambda PP resulted in a steep increase in scaffold availability, as shown by the rapid rebinding of the mExoS to the scaffold. The end values quickly leveled off at the same value as those of the unphosphorylated scaffold. This confirmed that full dephosphorylation of the 14-3-3 scaffold occurred, effectively and fully reverting the autoinhibition. Complete dephosphorylation was also confirmed by LC–MS analysis (Figure S15). Incubation with a lower concentration of Lambda PP (1.0 U/μL) led to the same end-point, but required more time (after ∼3 h). The rate of scaffold reactivation can thus be tuned by the phosphatase activity.

## Conclusions

In conclusion, we presented a modular strategy to introduce reversible control over scaffold protein activity. The scaffold platform features the bivalent 14-3-3 protein as chassis, fused to regulatory peptide motifs that block the two 14-3-3 phospho-binding sites upon phosphorylation by specific kinases. Competitive binding assays showed clear autoinhibitory scaffolding effects upon the phosphorylation of the inhibitory motifs with the corresponding kinases. Phosphorylation of the *intra*molecular motifs induced up to a 35-fold decrease in binding affinity for *inter*molecular binding partners. Tuning of kinase concentration allowed control over the level of phosphorylation-induced displacement of the 14-3-3 interaction partners. Engineering of the appended peptide motifs allowed the regulation of phosphorylation and autoinhibition by different kinases, as well as dual kinase input regulation. Reversibility of the autoinhibition was easily achieved by the enzymatic dephosphorylation of the scaffold platform, resulting in full recovery of scaffold activity.

The modularity of this scaffold protein platform allows the exchange of peptide motifs for tuning of autoinhibition affinity and valency, as well as tailoring for responsivity to a specific kinase of interest, provided that corresponding peptide motifs with sufficient binding affinity are available.^19,20^ Interestingly, the chemical variety that can be achieved with these systems will also allow to study the potential relevance of an interplay between the affinity and kinetics of the 14-3-3 binding moieties, with the Michaelis–Menten properties of the kinase at hand. The robust protein architecture permits fusion of peptides to the N- and C-termini, enabling the engineering of scaffolds with (a)symmetrical combinations of regulatory peptides to further implement logic operations such as OR-gating. A combination with other synthetic signaling elements,^5^ such as (small molecule-gated) split-kinases and phosphatases, could be harnessed to introduce feedback control to these synthetic signaling networks.

Natural examples of intramolecular regulation of scaffold proteins by phosphorylation typically entail scaffold activation by phosphorylation.^8,9,11^ Our approach conceptually opens up routes for reversed mechanisms; that is, the inhibition of scaffold function upon the activation of kinase signaling pathways. Interestingly, the dual-input regulation concept would allow to ensure the need for two different kinase signaling pathways to be active. We envision these engineered scaffolds to also offer entries as plug-and-play elements to introduce enhanced control over 14-3-3-templated PPIs in natural signaling networks, potentially by replacing natural isoforms of 14-3-3 by these auto-inhibitory constructs. This would complement eukaryotic cells with either replaced 14-3-3 or additional 14-3-3 scaffold platforms.^28^ The scaffolds could, for instance, be employed to modulate and study downstream signaling processes either activated or inhibited by the regulatory 14-3-3 platform. Decorating proteins of interest with 14-3-3 recognition motifs would also allow to generate non-native 14-3-3-templated binding and thus allow to reversibly control protein (co-)localization and PPIs on these synthetic platforms. Especially, the control over the activity of (dimeric) enzymes is attractive to be further explored with these modular 14-3-3 scaffolding platforms.

## Materials and Methods

### Plasmid Construction

Restriction enzymes, polymerases, ligases, and reaction buffers were acquired from New England Biolabs. Primers for cloning were ordered at Integrated DNA Technologies. A pET28a plasmid encoding for dT14-3-3 fused to a biExoS peptide with a TEV-cleavable GGS linker was used as the basis of the cloning procedures.^25^ gBlocks encoding for inhibitory peptide motifs biPKB, biCHK1, biPKA, PKA/PKB, PKA/CHK1, and PKB/CHK1 were synthesized by Integrated DNA Technologies.

A KpnI restriction site was first introduced adjacent to the undesired peptide sequence via site-directed mutagenesis (QuikChange Lightning Multi Site-Directed mutagenesis kit, Agilent). All gBlocks were amplified to introduce matching KpnI and HindIII restriction sites adjacent to the sequences to be ligated into the plasmid. Plasmid and gBlock DNA sequences were then double-digested with KpnI and HindIII and purified using the Qiaquick Gel Extraction kit (Qiagen) and the Qiaquick PCR Purification kit (Qiagen), respectively. Finally, the digested gBlocks were ligated into the linearized plasmid using T4 DNA ligase. All DNA sequences were verified by Sanger dideoxy sequencing (BaseClear).

### Protein Expression and Purification

Plasmids were transformed into *E. coli* BL21(DE3) cells (Novagen) via a 30-second heat shock at 42 °C, followed by incubation in SOC medium at 37 °C and 300 rpm for 30 min. Subsequently, the cells were used to inoculate 8 mL of Luria-Bertani (LB) medium cultures supplemented with 30 μg/mL kanamycin, shaking overnight at 37 °C and 250 rpm. The cell cultures were then added to 2 L of LB medium supplemented with 30 μg/mL kanamycin and incubated at 37 °C and 150 rpm. When the optical density OD_600_ reached 0.6, protein expression was induced using 0.5 mM isopropyl-β-D-thiogalactopyranoside (IPTG). Following overnight expression at 18 °C and 160 rpm, cells were harvested by centrifugation at 4 °C and 10,000×*g* for 10 min. The resulting cell pellets were resuspended in 10 mL/g_cell pellet_ lysis buffer supplemented with 1 μL/g_cell pellet_ benzonase nuclease (Novagen), after which, cells were lysed by three runs through an EmulsiFlexC3 high pressure homogenizer (Avestin) at 15,000 psi. Cell debris was removed by centrifugation at 4 °C and 40,000×*g* for 20 min, after which the supernatants were applied to Ni-loaded columns (His-Bind Resin, Novagen). Following the application of wash buffer B and wash buffer A to remove nonspecifically bound products, the proteins were eluted from the columns using elution buffer. The collected protein fractions were concentrated using Amicon Ultra Centrifugal Filters (10 kDa MWCO, Millipore) and buffer-exchanged into assay buffer using PD-10 desalting columns (GE Healthcare). Protein concentrations were determined via absorption measurements at 280 nm using an ND-1000 spectrophotometer (Thermo Scientific) and calculated extinction coefficients in water under reducing conditions. Aliquots of the proteins were frozen in liquid nitrogen for storage at −80 °C. Protein mass and purity were verified via SDS-PAGE and Q-ToF LC–MS analysis.

### Q-ToF LC–MS Analysis

Mass and purity of the proteins and peptides were determined using a high-resolution Q-ToF LC–MS system consisting of a ACQUITY UPLC I-Class system (Waters) coupled to a Xevo G2 quadrupole time of flight. The system comprised a Binary Solvent Manager and a Sample Manager with Fixed-Loop (SM-FL). The protein was separated (0.3 mL min^–1^) on a column (Polaris C18A reverse phase column 2.0 × 100 mm, Agilent) using a 15–75% acetonitrile gradient in water supplemented with 0.1% v/v formic acid before analysis in positive mode in the mass spectrometer. The *m*/*z* spectra were deconvoluted using the MaxENTI algorithm in the Masslynx v4.1 (SCN862) software.

### Fluorescence Polarization Assays

FITC-labeled peptides were dissolved in assay buffer supplemented with 0.1% Tween20 to a final concentration of 15 nM. Two-fold dilution series of protein were made in black, round-bottom 384-well plates (Corning) in a final volume of 20 μL in triplicates. Fluorescence polarization was measured with a Tecan Infinite F500 plate reader at room temperature (filter set λ_ex_: 485 ± 20 nm, λ_em_: 525 ± 25 nm). The gain was optimized for each peptide, and the G-factor was optimized using blank measurements (buffer only) and reference (peptide only). Plotted polarization values are the means of three measurements with standard error bars. Dissociation constants were determined by fitting using a one site-specific binding model (eq 1).
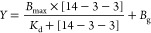
1Here, *B*_max_ is the maximum binding in the same unit as *Y*, [14-3-3] is the concentration of 14-3-3 monomer, *K*_d_ is the equilibrium dissociation constant, and *B*_g_ is the polarization value in the absence of 14-3-3.

### In Vitro Phosphorylation Assay

For Q-ToF LC–MS analysis, 40 μM of each protein construct was phosphorylated by incubation with the corresponding kinase(s) PKA (2 μL 2500 U/μL, New England Biolabs), PKB (2 μL 10.3 U/μL, Sigma-Aldrich), and CHK1 (5 μL 16.9 U/μL, Sigma-Aldrich) in the presence of ATP (500 μM) in phosphorylation buffer at 30 °C, 300 rpm for 16 h (total volume of 100 μL). Subsequently, the reactions were diluted in water (22 μM, total volume 100 μL) supplemented with TEV protease (2 μL 19.32 mg/mL, purified in-house) to cleave dT14-3-3 from its inhibitory peptide sequences. Following incubation at 30 °C, 300 rpm for 3 h, the reaction buffer was changed to water with 0.1% formic acid for Q-ToF LC–MS analysis.

For fluorescence polarization assays, protein constructs dT14-3-3_biPKB, dT14-3-3_biCHK1, and dT14-3-3_biPKA (66.7 μM) were phosphorylated by incubation with their corresponding kinases PKA (5 μL 2500 U/μL, New England Biolabs), PKB (5 μL 10.3 U/μL, Sigma-Aldrich), CHK1 (10 μL 16.9 U/μL, Sigma-Aldrich) in the presence of ATP (500 μM) in phosphorylation buffer at 30 °C, 300 rpm for 16 h (total volume 300 μL). Subsequent fluorescence polarization measurements were performed in the same manner as described above.

### Kinetic In Vitro Phosphorylation Assay

A 2× mastermix was prepared by incubating each bivalent protein construct (10 μM for interaction with mExoS, 80 nM for the interaction with biExoS) in the presence of an FITC-labeled ExoS peptide (30 nM), MgCl_2_ (40 mM) and ATP (1 mM) in assay buffer supplemented with 0.2% Tween20 (total volume 220 μL). Serial dilutions of 2× PKA (90–3.3 U/μL), 2× PKB (0.8–0.04 U/μL), and 2× CHK1 (1.4–0.06 U/μL) were prepared in assay buffer. Subsequently, 10 μL of 2× Mastermix was mixed with 10 μL of each corresponding 2× kinase dilution in black, round-bottom 384-well plates (Corning) in triplicates. Plates were directly sealed (Greiner EASYseal), and kinetic fluorescence polarization measurements were performed with a Tecan Infinite F500 platereader at 30 °C for 10 h (filter set λ_ex_: 485 ± 20 nm, λ_em_: 525 ± 25 nm). The gain was optimized for each peptide, and the G-factor was optimized using blank measurements (buffer only) and reference (peptide only). Plotted polarization values over time are the means of three measurements with standard error clouds.

### Kinetic In Vitro Dephosphorylation Assay

dT14-3-3_biPKA (40 μM) was incubated in the presence of PKA (3 μL 2500 U/μL, New England Biolabs) and ATP (500 μM) in phosphorylation buffer at 30 °C, 300 rpm for 5 h (total volume 70 μL). Subsequently, the phosphorylated protein was diluted to 10 μM in assay buffer containing 0.2% Tween20, supplemented with FITC-labeled mExoS (30 nM) and H89 (100 μM) (total volume 220 μL). Lambda protein phosphatase (New England Biolabs, 400 U/μL) was diluted to 20 U/μL and 2 U/μL in assay buffer supplemented with MnCl2 (20 mM) (total volume 20 μL). Phosphorylated protein (10 μL) was mixed with 10 μL of each phosphatase dilution in black, round-bottom 384-well plates (Corning) in triplicates. The plate was directly sealed (Greiner EASYseal), and kinetic fluorescence polarization measurements were performed with a Tecan Infinite F500 platereader at 30 °C for 10 h (filter set λ_ex_: 485 ± 20 nm, λ_em_: 525 ± 25 nm). The gain was optimized, and the G-factor was optimized using blank measurements (buffer only) and reference (peptide only). Plotted polarization values over time are the means of three measurements with standard error clouds.

## Supporting Information Available

Experimental details, Supporting Figures S1–S15, Supporting Tables S1–S4 (PDF)
