# Supplementary material for: Switchable Control of Scaffold Protein Activity via Engineered Phosphoregulated Autoinhibition
Source: ACS Synth Biol. 2022 Jun 29;11(7):2464–72. doi: 10.1021/acssynbio.2c00122 (PMC9295147; doi:10.1021/acssynbio.2c00122)
Supplement: Supplementary file 1 — sb2c00122_si_001.pdf [file sb2c00122_si_001.pdf]

Supporting Information for

Switchable control of scaffold protein activity via engineered phosphoregulated autoinhibition.

Arjan Hazegh Nikroo,[#] Lenne J. M. Lemmens,[#] Tim Wezeman, Christian Ottmann, Maarten Merkx, Luc Brunsveld*

AUTHOR ADDRESS

Laboratory of Chemical Biology, Department of Biomedical Engineering and Institute for Complex Molecular Systems, Technische Universiteit Eindhoven, Den Dolech 2, Eindhoven, 5612 AZ, The Netherlands.

Contents

Buffers S3

Sequences of protein constructs S3

Supporting figures S5

Figure S1 | Schematic overview of plasmid composition and cloning strategy S5

Figure S2 | Crystal structure of a 14-3-3ζ dimer bound to a c-Raf peptide. S5

Figure S3 | Crystal structure of a 14-3-3ζ dimer bound to CDC25C peptides S5

Figure S4 | Q-ToF LC-MS analysis of synthesized phosphorylated peptides. S6

Figure S5 | Fluorescence polarization assay of phosphorylated monovalent peptides with dT14-3-3. .... S6

Figure S6 |Crystal structure of a T14-3-3c monomer S6

Figure S7 | SDS-PAGE analysis of purified proteins S7

Figure S8 | Q-ToF LC-MS analysis of dT14-3-3. .... S7

Figure S9 | Q-ToF LC-MS analysis of phosphoregulated dT14-3-3 constructs S8

Figure S10 | Schematic representation of TEV protease-mediated linker cleavage S9

Figure S11 | Q-ToF LC-MS analysis of bivalent phosphoregulated dT14-3-3 constructs after cleavage S9

Figure S12 | Q-ToF LC-MS analysis of hetero-bivalent phosphoregulated dT14-3-3 constructs after cleavage S10

Figure S13 | Fluorescence polarization assays of ExoS peptides with phosphoregulated dT14-3-3 constructs S11

Figure S14 | Q-ToF LC-MS analysis of phosphorylated dT14-3-3_biPKA after addition of H89 and linker cleavage S11

Figure S15 | Q-ToF LC-MS analysis of dT14-3-3_biPKA after dephosphorylation and linker cleavage S12

Supporting tables S12

Table S1 | Overview of identified bivalent peptides S12

Table S2 | Overview of FITC-labelled peptides used in titration experiments S12

Table S3 | Overview of FITC-labelled ExoS peptides S12

Table S4 | Conversion table for kinase stock concentrations S12

References S13

Buffers

Lysis buffer: 50 mM Tris, 300 mM NaCl, 50 mM imidazole, 5 mM $MgCl_2$, pH 8.0.
Wash buffer A: 50 mM Tris, 300 mM NaCl, 50 mM imidazole, 0.1% Triton-X-100, pH 8.0.
Wash buffer B: 50 mM Tris, 300 mM NaCl, 50 mM imidazole, pH 8.0.
Elution buffer: 50 mM Tris, 300 mM NaCl, 250 mM imidazole, pH 8.0.
Assay buffer: 10 mM HEPES, 150 mM NaCl, pH 7.4.
Phosphorylation buffer: 10 mM HEPES, 150 mM NaCl, 20 mM $MgCl_2$, pH 7.4.

Sequences of protein constructs

- (green) = His_6-tag
- (dark grey) = N-terminal T14-3-3 monomer
- (light grey) = C-terminal T14-3-3 monomer
- (red) = TEV protease cleavage site
- (yellow) = Inhibitory peptide
- (magenta) = Strep-tag

dT14-3-3cΔC (dT14-3-3)

```
  1   MGGSHHHHHH GTGASSSGMA VAPTAREENV YMAKLAEQAE RYEEMVEFME KVSNSLGSEE   60
 61   LTVEERNLLS VAYKNVIGAR RASWRIISSI EQKEESRGNE EHVNSIREYR SKIENELSKI  120
121   CDGILKLLDA KLIPSAASGD SKVFYLKMKG DYHRYLAEFK TGAERKEAAE STLTAYKAAQ  180
181   DIATTELAPT HPIRLGLALN FSVFYYEILN SPDRACNLAK QAFDEAIAEL DTLGEESYKD  240
241   STLIMQLLRD NLTLWTSDMQ GGSGGSGGSG GSGGSGGSGG SGGSGGSGGS MAVAPTAREE  300
301   NVYMAKLAEQ AERYEEMVEF MEKVSNSLGS EELTVEERNL LSVAYKNVIG ARRASWRIIS  360
361   SIEQKEESRG NEEHVNSIRE YRSKIENELS KICDGILKLL DAKLIPSAAS GDSKVFYLKM  420
421   KGDYHRYLAE FKTGAERKEA AESTLTAYKA AQDIATTELA PTHPIRLGLA LNFSVFYYEI  480
481   LNSPDRACNL AKQAFDEAIA ELDTLGEESY KDSTLIMQLL RDNLTLWTSD MQGTGGNGSS  540
541   WSHPQFEKGG S                                                   551
```

dT14-3-3-cRaf_S233/S259 (dT14-3-3_biPKA)

```
  1   MGSSHHHHHH SSGLVPRGSH MAVAPTAREE NVYMAKLAEQ AERYEEMVEF MEKVSNSLGS   60
 61   EELTVEERNL LSVAYKNVIG ARRASWRIIS SIEQKEESRG NEEHVNSIRE YRSKIENELS  120
121   KICDGILKLL DAKLIPSAAS GDSKVFYLKM KGDYHRYLAE FKTGAERKEA AESTLTAYKA  180
181   AQDIATTELA PTHPIRLGLA LNFSVFYYEI LNSPDRACNL AKQAFDEAIA ELDTLGEESY  240
241   KDSTLIMQLL RDNLTLWTSD MQGGSGGSGG SGGSGGSGGS GGSGGSGGSG GSMAVAPTAR  300
301   EENVYMAKLA EQAERYEEMV EFMEKVSNSL GSEELTVEER NLLSVAYKNV IGARRASWRI  360
361   ISSIEQKEES RGNEEHVNSI REYRSKIENE LSKICDGILK LLDAKLIPSA ASGDSKVFYL  420
421   KMKGDYHRYL AEFKTGAERK EAAESTLTAY KAAQDIATTE LAPTHPIRLG LALNFSVFYY  480
481   EILNSPDRAC NLAKQAFDEA IAELDTLGEE SYKDSTLIMQ LLRDNLTLWT SDMQGTSGGS  540
541   GGSGGSGGSD DVTPCSMSGG SGGSENLYFQ SGGSGGSGGS GGSGGSGGSG GSGGSGALSG  600
601   GSTGTQHRYS TPHAFTFNTS SPSSEGSLSQ RQRSTSTPNV HGGSGGSGGS WSHPQFEK    658
```

dT14-3-3-biGSK3a_S21 (dT14-3-3_biPKB)

```
  1   MGSSHHHHHH SSGLVPRGSH MAVAPTAREE NVYMAKLAEQ AERYEEMVEF MEKVSNSLGS   60
 61   EELTVEERNL LSVAYKNVIG ARRASWRIIS SIEQKEESRG NEEHVNSIRE YRSKIENELS  120
121   KICDGILKLL DAKLIPSAAS GDSKVFYLKM KGDYHRYLAE FKTGAERKEA AESTLTAYKA  180
181   AQDIATTELA PTHPIRLGLA LNFSVFYYEI LNSPDRACNL AKQAFDEAIA ELDTLGEESY  240
241   KDSTLIMQLL RDNLTLWTSD MQGGSGGSGG SGGSGGSGGS GGSGGSGGSG GSMAVAPTAR  300
301   EENVYMAKLA EQAERYEEMV EFMEKVSNSL GSEELTVEER NLLSVAYKNV IGARRASWRI  360
361   ISSIEQKEES RGNEEHVNSI REYRSKIENE LSKICDGILK LLDAKLIPSA ASGDSKVFYL  420
421   KMKGDYHRYL AEFKTGAERK EAAESTLTAY KAAQDIATTE LAPTHPIRLG LALNFSVFYY  480
481   EILNSPDRAC NLAKQAFDEA IAELDTLGEE SYKDSTLIMQ LLRDNLTLWT SDMQGTSGGS  540
541   GGSGGSGGSD DVTPCSMSGG SGGSENLYFQ SGGSGGSGGS GGSGGSGGSG GSGGSGALSG  600
601   GSTGTRARTS SFAEPGGSGG SGGSGSRART SSFAEPGGSG GSGGSWSHPQ FEK          653
```

dT14-3-3-biCDC25C_S216 (dT14-3-3_biCHK1)

```
  1   MGSSHHHHHH SSGLVPRGSH MAVAPTAREE NVYMAKLAEQ AERYEEMVEF MEKVSNSLGS   60
```

```
 61  EELTVEERNL LSVAYKNVIG ARRASWRIIS SIEQKEESRG NEEHVNSIRE YRSKIENELS  120
121  KICDGILKLL DAKLIPSAAS GDSKVFYLKM KGDYHRYLAE FKTGAERKEA AESTLTAYKA  180
181  AQDIATTELA PTHPIRLGLA LNFSVFYYEI LNSPDRACNL AKQAFDEAIA ELDTLGEESY  240
241  KDSTLIMQLL RDNLTLWTSD MQGGSGGSGG SGGSGGSGGS GGSGGSGGSG GSMAVAPTAR  300
301  EENVYMAKLA EQAERYEEMV EFMEKVSNSL GSEELTVEER NLLSVAYKNV IGARRASWRI  360
361  ISSIEQKEES RGNEEHVNSI REYRSKIENE LSKICDGILK LLDAKLIPSA ASGDSKVFYL  420
241  KMKGDYHRYL AEFKTGAERK EAAESTLTAY KAAQDIATTE LAPTHPIRLG LALNFSVFYY  480
481  EILNSPDRAC NLAKQAFDEA IAELDTLGEE SYKDSTLIMQ LLRDNLTLWT SDMQGTSGGS  540
541  GGSGGSGGSD DVTPCSMSGG SGGSENLYFQ SGGSGGSGGS GGSGGSGGSG GSGGSGALSG  600
601  GSTGTLYRSP SMPENGGSGG SGGSGSLYRS PSMPENGGSG GSGGSWSHPQ FEK         653
```

dT14-3-3-cRaf_S259-GSK3a_S21 (dT14-3-3_PKA/PKB)

```
  1  MGSSHHHHHH SSGLVPRGSH MAVAPTAREE NVYMAKLAEQ AERYEEMVEF MEKVSNSLGS   60
 61  EELTVEERNL LSVAYKNVIG ARRASWRIIS SIEQKEESRG NEEHVNSIRE YRSKIENELS  120
121  KICDGILKLL DAKLIPSAAS GDSKVFYLKM KGDYHRYLAE FKTGAERKEA AESTLTAYKA  180
181  AQDIATTELA PTHPIRLGLA LNFSVFYYEI LNSPDRACNL AKQAFDEAIA ELDTLGEESY  240
241  KDSTLIMQLL RDNLTLWTSD MQGGSGGSGG SGGSGGSGGS GGSGGSGGSG GSMAVAPTAR  300
301  EENVYMAKLA EQAERYEEMV EFMEKVSNSL GSEELTVEER NLLSVAYKNV IGARRASWRI  360
361  ISSIEQKEES RGNEEHVNSI REYRSKIENE LSKICDGILK LLDAKLIPSA ASGDSKVFYL  420
421  KMKGDYHRYL AEFKTGAERK EAAESTLTAY KAAQDIATTE LAPTHPIRLG LALNFSVFYY  480
481  EILNSPDRAC NLAKQAFDEA IAELDTLGEE SYKDSTLIMQ LLRDNLTLWT SDMQGTSGGS  540
541  GGSGGSGGSD DVTPCSMSGG SGGSENLYFQ SGGSGGSGGS GGSGGSGGSG GSGGSGALSG  600
601  GSTGTRQRST STPNVGGSGG SGGSGSRART SSFAEPGGSG GSGGSWSHPQ FEK         653
```

dT14-3-3-cRaf_S259-CDC25C_S216 (dT14-3-3_PKA/CHK1)

```
  1  MGSSHHHHHH SSGLVPRGSH MAVAPTAREE NVYMAKLAEQ AERYEEMVEF MEKVSNSLGS   60
 61  EELTVEERNL LSVAYKNVIG ARRASWRIIS SIEQKEESRG NEEHVNSIRE YRSKIENELS  120
121  KICDGILKLL DAKLIPSAAS GDSKVFYLKM KGDYHRYLAE FKTGAERKEA AESTLTAYKA  180
181  AQDIATTELA PTHPIRLGLA LNFSVFYYEI LNSPDRACNL AKQAFDEAIA ELDTLGEESY  240
241  KDSTLIMQLL RDNLTLWTSD MQGGSGGSGG SGGSGGSGGS GGSGGSGGSG GSMAVAPTAR  300
301  EENVYMAKLA EQAERYEEMV EFMEKVSNSL GSEELTVEER NLLSVAYKNV IGARRASWRI  360
361  ISSIEQKEES RGNEEHVNSI REYRSKIENE LSKICDGILK LLDAKLIPSA ASGDSKVFYL  420
421  KMKGDYHRYL AEFKTGAERK EAAESTLTAY KAAQDIATTE LAPTHPIRLG LALNFSVFYY  480
481  EILNSPDRAC NLAKQAFDEA IAELDTLGEE SYKDSTLIMQ LLRDNLTLWT SDMQGTSGGS  540
541  GGSGGSGGSD DVTPCSMSGG SGGSENLYFQ SGGSGGSGGS GGSGGSGGSG GSGGSGALSG  600
601  GSTGTRQRST STPNVGGSGG SGGSGSLYRS PSMPENGGSG GSGGSWSHPQ FEK         653
```

dT14-3-3-GSK3a_S21-CDC25C_S216 (dT14-3-3_PKB/CHK1)

```
  1  MGSSHHHHHH SSGLVPRGSH MAVAPTAREE NVYMAKLAEQ AERYEEMVEF MEKVSNSLGS   60
 61  EELTVEERNL LSVAYKNVIG ARRASWRIIS SIEQKEESRG NEEHVNSIRE YRSKIENELS  120
121  KICDGILKLL DAKLIPSAAS GDSKVFYLKM KGDYHRYLAE FKTGAERKEA AESTLTAYKA  180
181  AQDIATTELA PTHPIRLGLA LNFSVFYYEI LNSPDRACNL AKQAFDEAIA ELDTLGEESY  240
241  KDSTLIMQLL RDNLTLWTSD MQGGSGGSGG SGGSGGSGGS GGSGGSGGSG GSMAVAPTAR  300
301  EENVYMAKLA EQAERYEEMV EFMEKVSNSL GSEELTVEER NLLSVAYKNV IGARRASWRI  360
361  ISSIEQKEES RGNEEHVNSI REYRSKIENE LSKICDGILK LLDAKLIPSA ASGDSKVFYL  420
421  KMKGDYHRYL AEFKTGAERK EAAESTLTAY KAAQDIATTE LAPTHPIRLG LALNFSVFYY  480
481  EILNSPDRAC NLAKQAFDEA IAELDTLGEE SYKDSTLIMQ LLRDNLTLWT SDMQGTSGGS  540
541  GGSGGSGGSD DVTPCSMSGG SGGSENLYFQ SGGSGGSGGS GGSGGSGGSG GSGGSGALSG  600
601  GSTGTRARTS SFAEPGGSGG SGGSGSLYRS PSMPENGGSG GSGGSWSHPQ FEK         653
```

Supporting figures

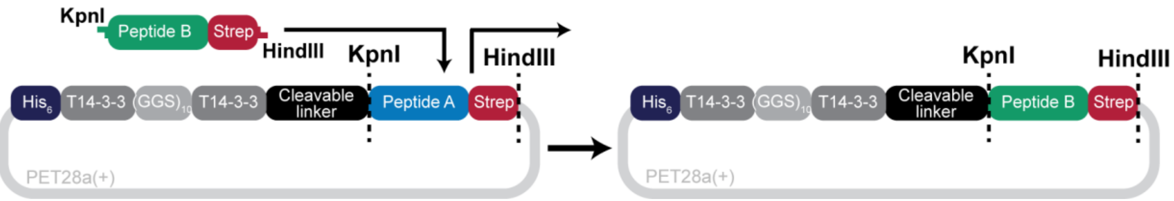

Figure S1 | Schematic overview of plasmid composition and cloning strategy. Restriction and ligation allows flexible exchange of peptide gBlock sequences.

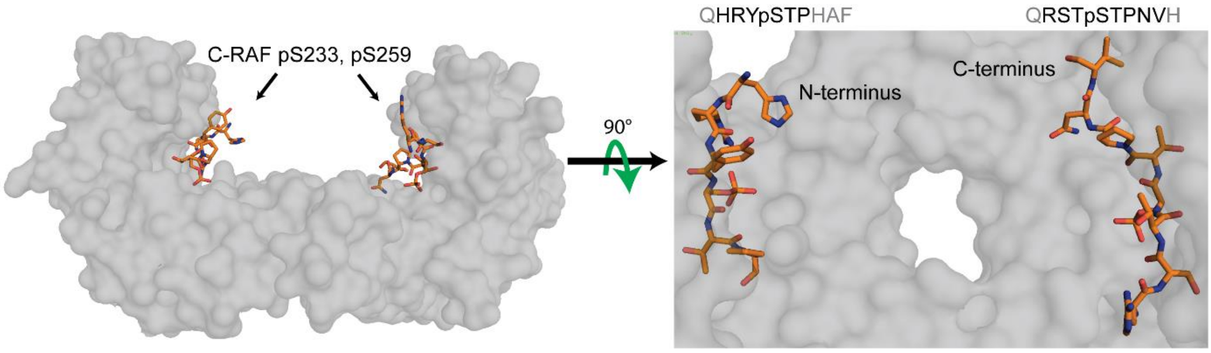

Figure S2 | Crystal structure of a 14-3-3ζ dimer bound to a c-Raf peptide. Front and top view of a 14-3-3ζ dimer (grey) bound by a c-Raf peptide containing two phosphorylated serine residues ps233, ps259 (orange). Letters in grey denote residues for which no electron density is observed. (PDB 4FJ3)

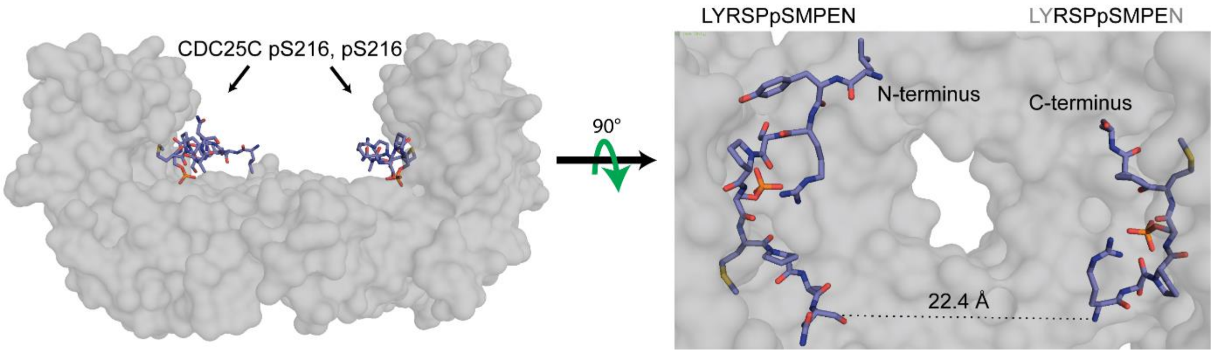

Figure S3 | Crystal structure of a 14-3-3ζ dimer bound to CDC25C peptides. Front and top view of a 14-3-3ζ dimer (grey) bound by CDC25C peptides containing phosphorylated serine residue ps216 (purple). Letters in grey denote residues for which no electron density is observed. Distance measurement between C- and N-termini of the two peptides reveals that the linker for the bivalent peptide, including residues L and Y, should at least span 22.4 Å. (PDB 5M35)

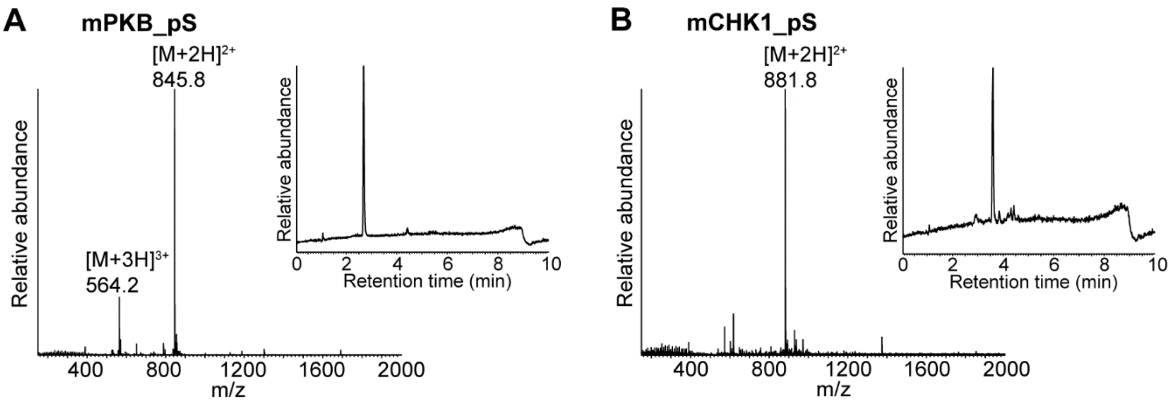

Figure S4 | Q-ToF LC-MS analysis of synthesized phosphorylated peptides. The m/z spectra are shown with the corresponding total ion count chromatogram as inlay. **A)** mPKB_pS. Calculated mass: 1690.7 Da, measured mass: 1689.6 Da **B)** mCHK1_pS. Calculated mass: 1762.8 Da, measured mass: 1761.6 Da.

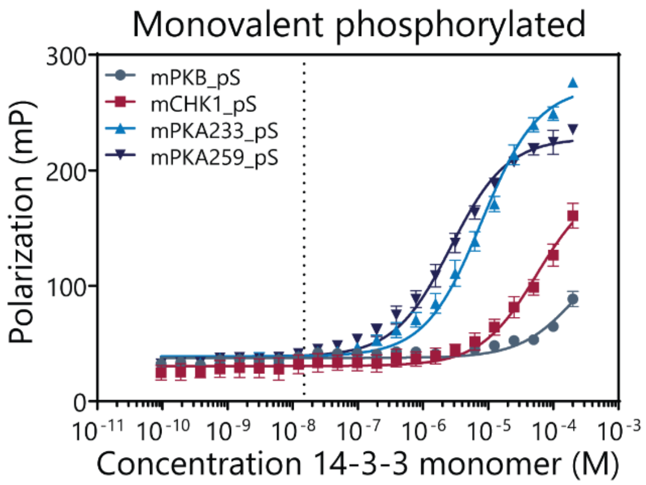

Peptide	K_d
mPKB_pS	Not measurable
mCHK1_pS	Not measurable
mPKA233_pS	7.96 ± 0.4 μM
mPKA259_pS	2.66 ± 0.1 μM

Figure S5 | Fluorescence polarization assay of phosphorylated monovalent peptides with dT14-3-3. dT14-3-3 was titrated (95 pM - 200 μM 14-3-3 monomer) to a fixed concentration of peptide (15 nM). The dashed line indicates stoichiometric amount of binding motif:14-3-3 monomer. Data fitting using Equation S1 resulted in the indicated binding affinities for mPKA233_pS and mPKA259_pS. Error bars represent SD (n=6).

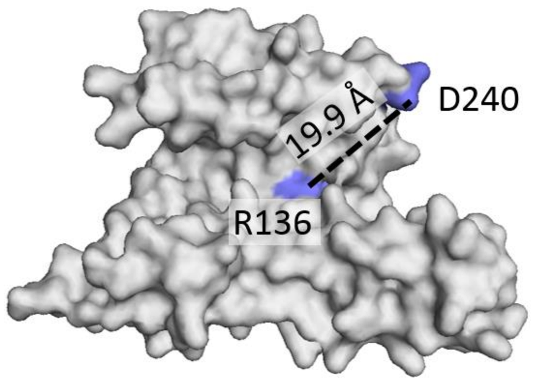

Figure S6 | Crystal structure of a T14-3-3c monomer. Distance measurement between residues R136 (left) and D240 (right) reveals a straight-line distance of 19.9 Å. (PDB 2O98)

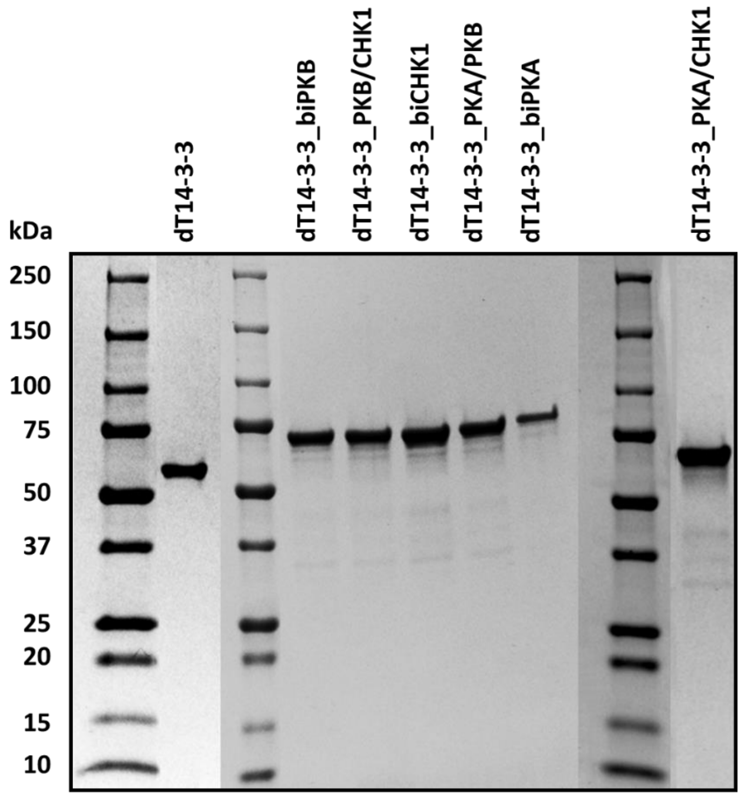

Figure S7| SDS-PAGE analysis of purified proteins. Expected masses: 69.1 kDa (dT14-3-3_biPKB), 69.2 kDa (dT14-3-3_biCHK1), 70.1 kDa (dT14-3-3_biPKA), 69.2 kDa (dT14-3-3_PKA/CHK1), 69.2 kDa (dT14-3-3_PKB/CHK1), 69.1 kDa (dT14-3-3_PKA/PKB). There are some very faint bands at lower molecular weight below the bands with anticipated molecular weight of the different proteins. The flexibly appended inhibitory peptide motifs might be slightly sensitive to truncation by bacterial proteases.

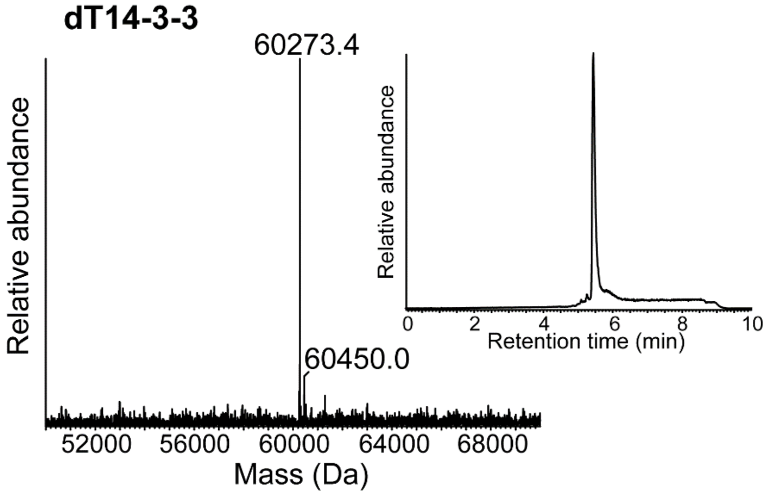

Figure S8 | Q-ToF LC-MS analysis of dT14-3-3. The deconvoluted mass spectrum is shown with the corresponding total ion count chromatogram as inlay. Expected masses: 60273.2 Da and 60451.2 (His_6-tag gluconoylation[1]).

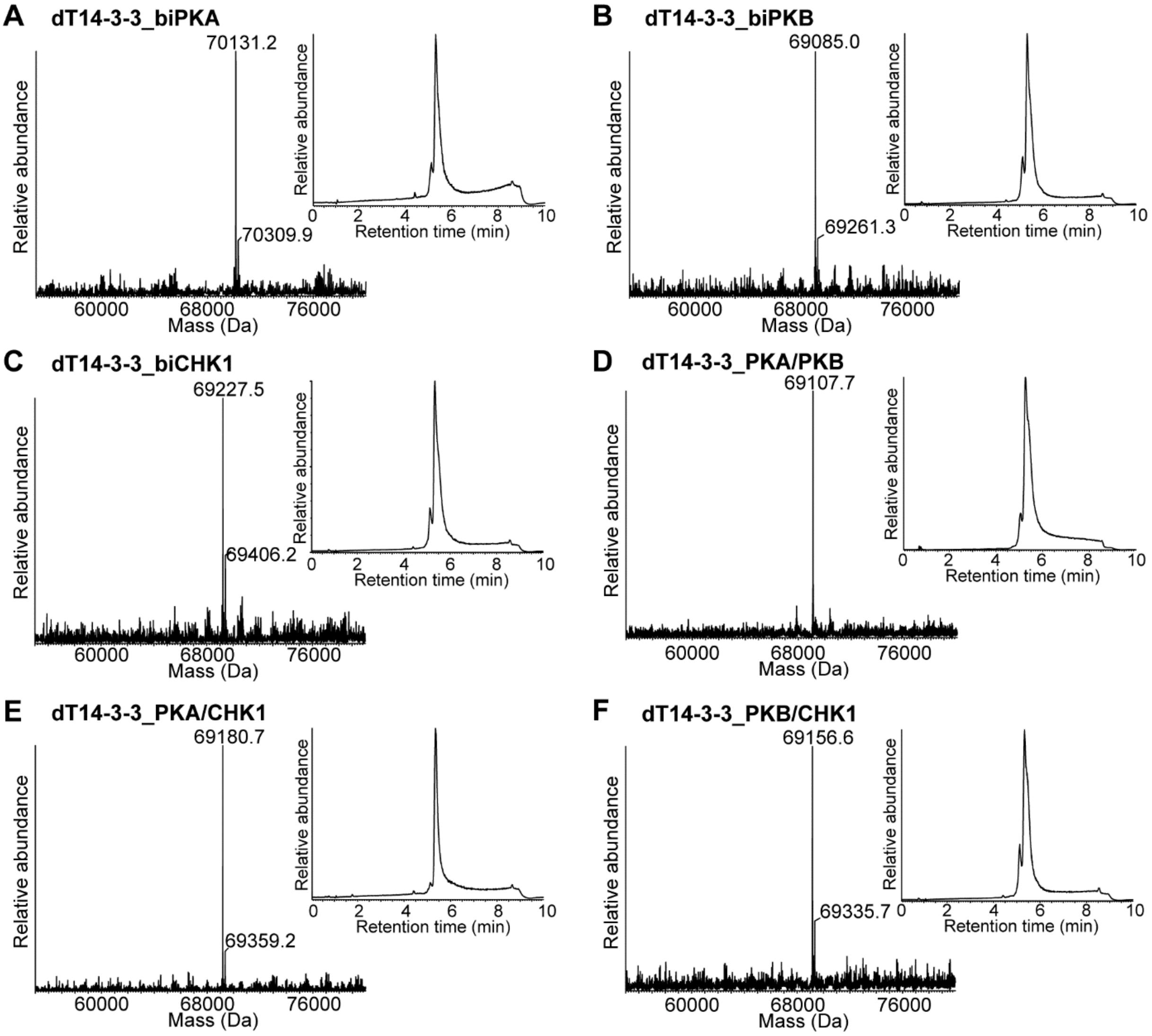

Figure S9 | Q-ToF LC-MS analysis of phosphoregulated dT14-3-3 constructs. The deconvoluted mass spectra are shown with the corresponding total ion count chromatogram as inlay. **A)** dT14-3-3_biPKA, expected mass: 70130.3 Da. **B)** dT14-3-3_biPKB, expected mass: 69084.2 Da. **C)** dT14-3-3_biCHK1, expected mass: 69228.4 Da. **D)** dT14-3-3_PKA/PKB, expected mass: 69108.3 Da. **E)** dT14-3-3_PKA/CHK1, expected mass: 69180.3 Da. **F)** dT14-3-3_PKB/CHK1, expected mass: 69156.3 Da. Additional indicated masses represent the expected mass including His_6-tag gluconoylation[1].

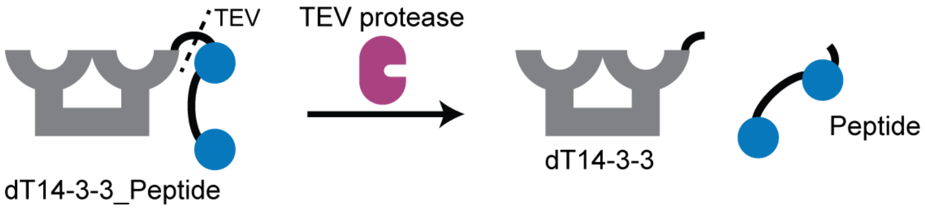

Figure S10 | Schematic representation of TEV protease-mediated linker cleavage. TEV protease cleaves the corresponding recognition motif (ENLYFQ$^{\nabla}$S) in the linker, enabling separate analysis of the dT14-3-3 protein moiety and a construct-specific inhibitory peptide.

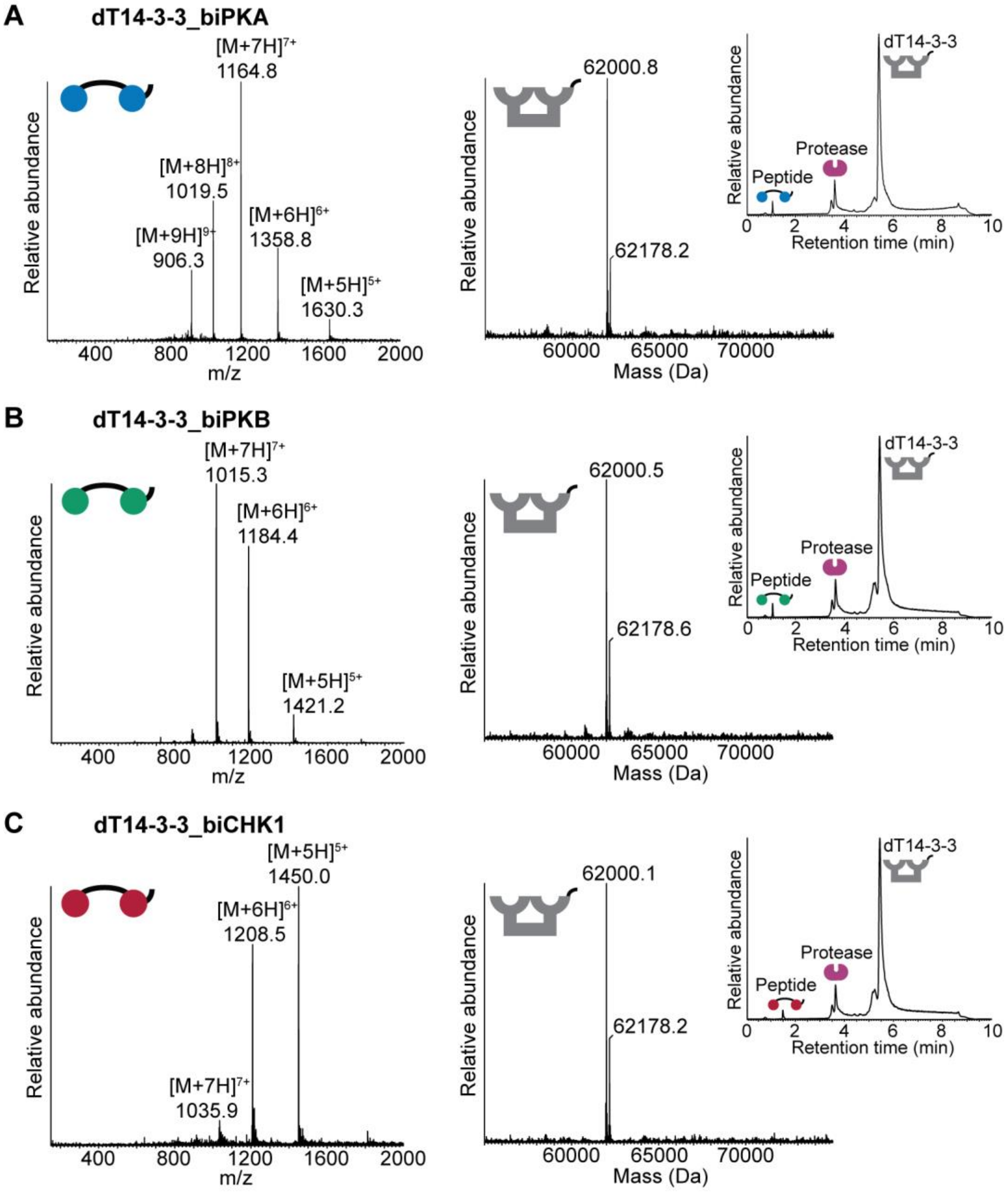

Figure S11 | Q-ToF LC-MS analysis of bivalent phosphoregulated dT14-3-3 constructs after cleavage. The m/z spectra of the peptide sequences (left) and deconvoluted mass spectra of dT14-3-3 (middle) are shown with the corresponding total ion count chromatograms as inlay. **A)** dT14-3-3_biPKA. Calculated mass peptide: 8147.3 Da, measured mass peptide: 8146.5 Da. **B)** dT14-3-3_biPKB. Calculated mass peptide: 7101.1 Da, measured mass peptide: 7100.0 Da. **C)** dT14-3-3_biCHK1. Calculated mass peptide: 7245.4 Da, measured mass peptide: 7245.0. Expected mass protein: 62001.1 Da and 62179.1 Da (His_6-tag gluconoylation[1]).

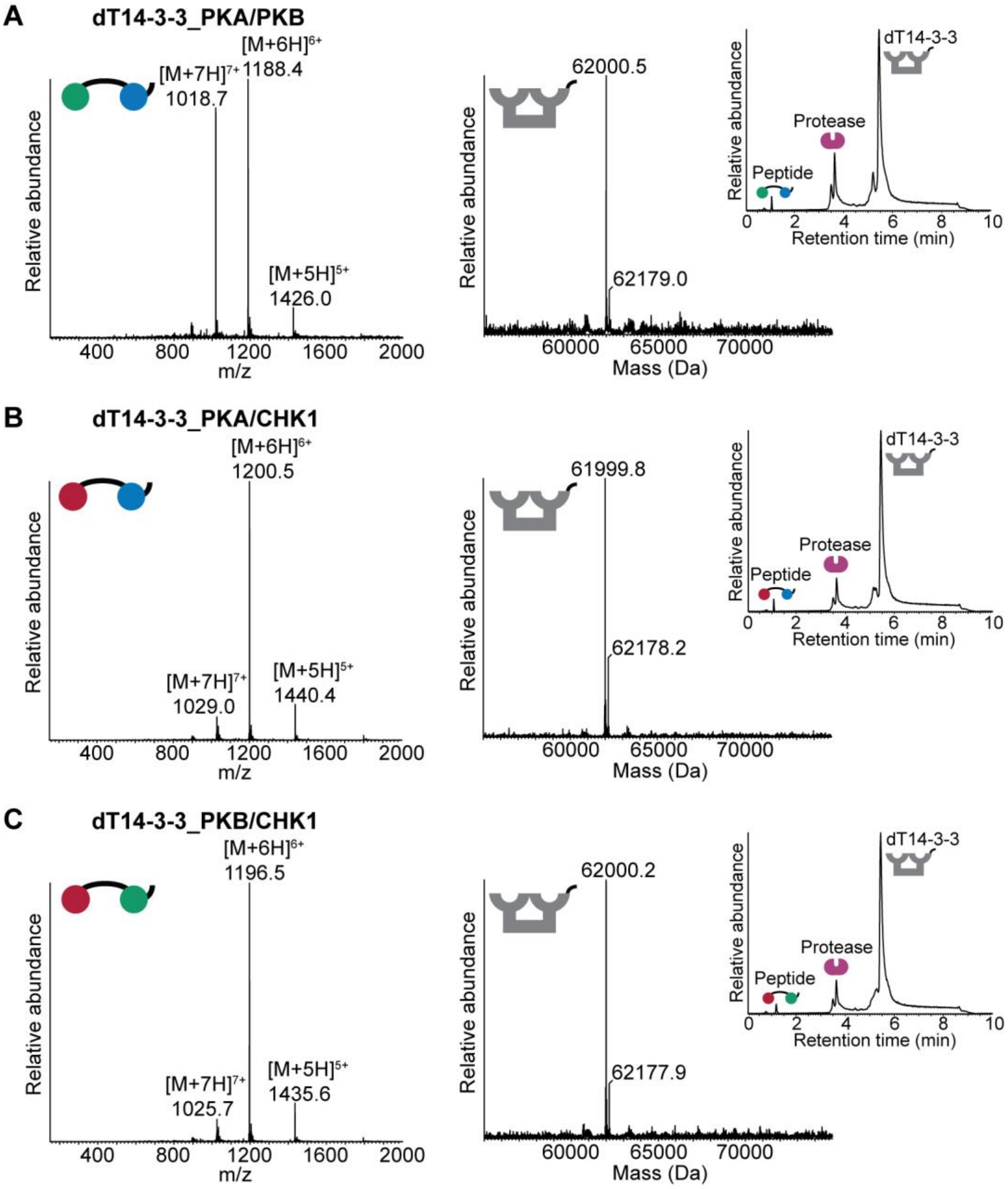

Figure S12 | Q-ToF LC-MS analysis of hetero-bivalent phosphoregulated dT14-3-3 constructs after cleavage. The m/z spectra of the peptide sequences (left) and deconvoluted mass spectra of dT14-3-3 (middle) are shown with the corresponding total ion count chromatograms as inlay. **A)** dT14-3-3_PKA/PKB. Calculated mass peptide: 7125.2 Da, measured mass peptide: 7124.4 Da. **B)** dT14-3-3_PKA/CHK1. Calculated mass peptide: 7197.3 Da, measured mass peptide: 7197.0 Da. **C)** dT14-3-3_PKB/CHK1. Calculated mass peptide: 7173.3 Da, measured mass peptide: 7173.0 Da. Expected mass protein: 62001.1 Da and 62179.1 Da (His_6-tag gluconoylation[1]).

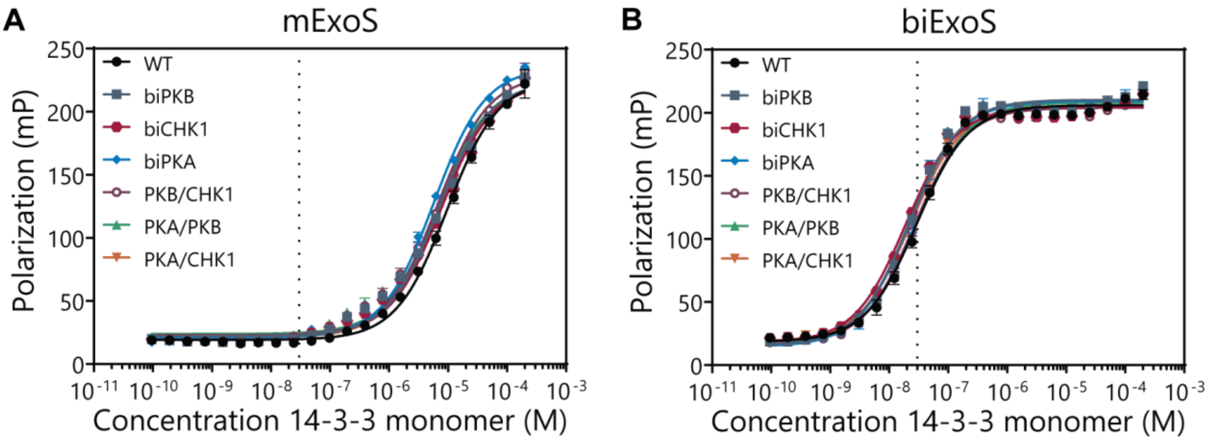

C

	mExoS	biExoS
**dT14-3-3 (WT)**	9.69 ± 0.36 µM	29.4 ± 1.4 nM
**dT14-3-3_biPKB**	6.91 ± 0.41 µM	22.0 ± 1.0 nM
**dT14-3-3_biCHK1**	7.57 ± 0.40 µM	18.9 ± 0.8 nM
**dT14-3-3_biPKA**	5.77 ± 0.19 µM	21.9 ± 1.3 nM
**dT14-3-3_PKB/CHK1**	6.62 ± 0.38 µM	26.3 ± 1.1 nM
**dT14-3-3_PKA/PKB**	6.43 ± 0.42 µM	29.1 ± 1.3 nM
**dT14-3-3_PKA/CHK1**	6.64 ± 0.33 µM	23.3 ± 0.9 nM

Figure S13 | Fluorescence polarization assays of ExoS peptides with phosphoregulated dT14-3-3 constructs. Protein constructs were titrated (95 pM - 200 µM 14-3-3 monomer) to a fixed concentration (15 nM) of FITC-labeled peptides **A)** mExoS, **B)** biExoS. The dashed line indicates stoichiometric amount of binding motif:14-3-3 monomer. **C)** Data fitting using Equation S1 (Experimental section) yielded the tabulated K_d values. Error bars represent SD (n=3).

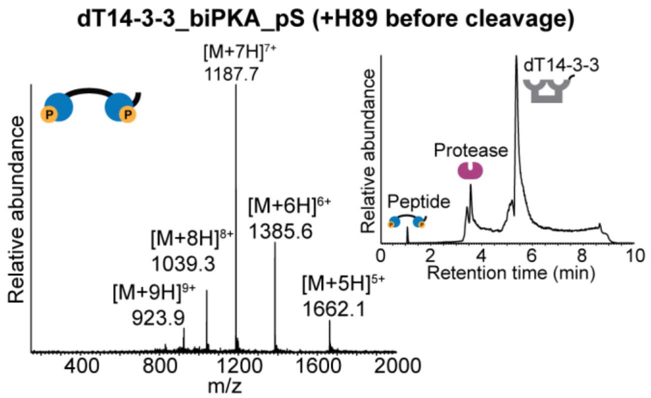

Figure S14 | Q-ToF LC-MS analysis of phosphorylated dT14-3-3_biPKA after addition of H89 and linker cleavage. To verify whether the observed phosphorylation occurred prior to cleavage of the flexible linker, dT14 3 3_biPKA was incubated with PKA, after which an excess of PKA inhibitor H89 was added to halt phosphorylation. Analysis following incubation with TEV protease revealed virtually identical spectra as compared to incubation without H89, illustrating that double phosphorylation occurred prior to incubation with TEV protease. The m/z spectrum of the peptide sequence is shown with the corresponding total ion count chromatogram as inlay. Calculated mass peptide: 8307.3 Da, measured mass peptide: 8306.8 Da.

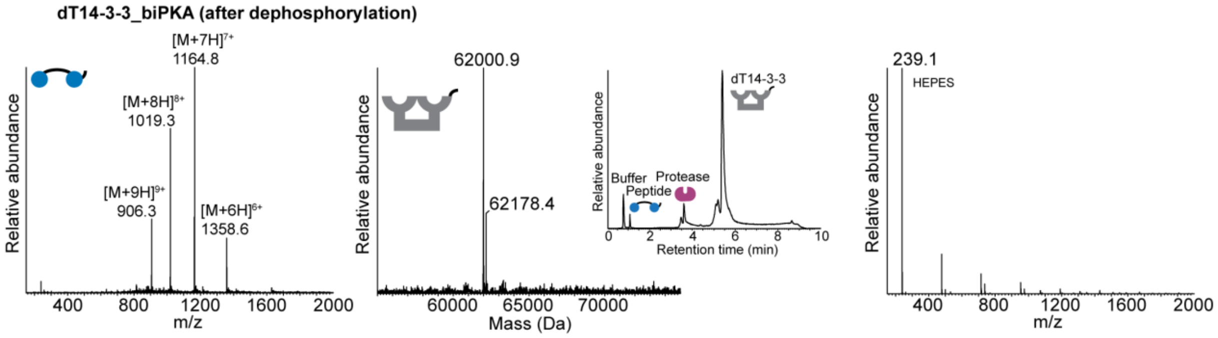

Figure S15 | Q-ToF LC-MS analysis of dT14-3-3_biPKA after dephosphorylation and linker cleavage. The m/z spectrum of the peptide (left) and deconvoluted mass spectrum of dT14-3-3 (middle) are shown with the corresponding total ion count chromatogram (inlay). The m/z spectrum corresponding to the buffer peak is shown right. Calculated mass biPKA peptide: 8147.3 Da, measured mass biPKA peptide: 8146.5. Expected mass dT14-3-3: 62001.1 Da and 62179.1 Da (His$_6$-tag gluconoylation[1]). Expected mass buffer component HEPES: 238.3 Da.

Supporting tables

Table S1 | Overview of identified bivalent peptides. Kinase recognition motifs are shown in bold, the putative phosphorylation sites are underlined and flexible linkers are shown in grey.

Name	Derived from	Sequence	Design
biPKA	c-Raf_S233/S259	**QHRYSTPHAF**TFNTSSPSSEGSLSQR**QRSTSTPNVH**	Homo-bival.
biCHK1	Cdc25C_biS216	**LYRSPSMPEN**GGSGGSGGSGS**LYRSPSMPEN**	Homo-bival.
biPKB	GSK3α_biS21	**RARTSSFAEP**GGSGGSGGSGS**RARTSSFAEP**	Homo-bival.
PKA/CHK1	c-Raf_S259/Cdc25C_S216	**RQRSTSTPNV**GGSGGSGGSGS**LYRSPSMPEN**	Hetero-bival.
PKA/PKB	c-Raf_S259/GSK3α_S21	**RQRSTSTPNV**GGSGGSGGSGS**RARTSSFAEP**	Hetero-bival.
PKB/CHK1	GSK3α_S21/Cdc25C_S216	**RARTSSFAEP**GGSGGSGGSGS**LYRSPSMPEN**	Hetero-bival.

Table S2 | Overview of FITC-labelled peptides used in titration experiment. Kinase recognition motifs are shown in bold, the putative phosphorylation sites are underlined and flexible linkers are shown in grey.

Name	Sequence
mPKA233_pS	FITC-6Ahx-**QHRYpSTPHAF**-NH$_2$
mPKA259_pS	FITC-6Ahx-**QRSTpSTPNVH**-NH$_2$
mCHK1_pS	FITC-O1Pen-**LYRSPpSMPEN**-NH$_2$
mPKB_pS	FITC-O1Pen-**RARTSpSFAEP**-NH$_2$

Table S3 | Overview of FITC-labeled ExoS peptides. The ExoS binding motif is shown in bold, while the flexible linkers are shown in grey.

Name	Sequence
mExoS	FITC-O1Pen-**QGLLDALDLAS**-NH$_2$
biExoS	FITC-6Ahx-**QGLLDALDLAS**GGGGGGGGGG**QGLLDALDLAS**-NH$_2$

Table S4 | Conversion table for kinase stock concentrations.

Kinase	Specific activity (U/mg)	MW (kDa)	Concentration (U/µL)	Concentration (µg/µL)	Concentration (µM)
PKA	~$5*10^6$	38	2500	0.5	13
PKB	~$0.10*10^6$	85	10.3	0.1	1.2
CHK1	~$0.17*10^6$	59	16.9	0.1	1.7

References

1 K. F. Geoghegan, H. B. F. Dixon, P. J. Rosner, L. R. Hoth, A. J. Lanzetti, K. A. Borzilleri, E. S. Marr, L. H. Pezzullo, L. B. Martin, P. K. Lemotte, A. S. McColl, A. V. Kamath and J. G. Stroh, *Anal. Biochem.*, 1999, **267**, 169–184.
